# Genome-wide identification of the *Hordeum vulgare* (L.) *CASPL* gene family and its diverse roles in response to drought and salt stress

**DOI:** 10.3389/fpls.2026.1966952

**Published:** 2026-09-29

**Authors:** Hui Sun, Xiangyu Meng, Yan Wang, Yongzhuo Guan

**Affiliations:** 1 College of Basic Medicine, Mudanjiang Medical University, Mudanjiang, China; 2 State Key Laboratory of Hybrid Rice, College of Life Sciences, Wuhan University, Wuhan, China

**Keywords:** drought and salt stresses, *HvCASPL* gene family, *HvCASPL5/16/22*, systematic evolution, TD and WGD

## Abstract

**Background:**

Casparian strip membrane domain proteins like (CASPL), exhibit profound associations with root development, stress responsiveness and mineral element uptake in plants. However, systematic characterization of the CASPL family in *H. vulgare* (*Hordeum vulgare*) and its involvement in drought and salt stresses remains unclear.

**Methods:**

In this study, a genome-wide identification and systematic analysis of the *HvCASPL* gene family was performed. Gene structure, conserved motifs, phylogenetic classification, duplication events, selective pressure, and cis-acting regulatory elements were comprehensively analyzed. Tissue-specific expression patterns and the expression responses of candidate *HvCASPL* genes to ABA, PEG6000-induced dehydration, and salt stress were further validated via RT-qPCR.

**Results:**

In this study, 36 *HvCASPL* genes were identified and classified into five Groups. Gene structure and conserved motif analyses revealed that 94% *HvCASPL* members possess the typical CASP domain, variations in exon-intron and motif composition suggested functional divergence. Tandem duplication (TD) and whole-genome duplication (WGD) were identified as the primary driving force for *HvCASPL* family expansion, with purifying selection (Ka/Ks<1) maintaining sequence integrity. Promoter cis-element analysis revealed a complex regulatory network involving hormone, stress responses, and growth and development signaling. Tissue expression patterns showed that *HvCASPL* genes were significantly differentially expressed in different tissues, with *HvCASPL12/19/22/23* showing higher expression levels in roots. Notably, RT-qPCR expression profiling on 14 selected genes from 36 *HvCASPL* family members demonstrated that eight *HvCASPL* genes (*HvCASPL5*, *HvCASPL16*, and *HvCASPL22*) were significantly upregulated in response to ABA, PEG6000 and salt treatment.

**Conclusions:**

These transcriptomic findings indicate that the *HvCASPL* family has undergone functional diversification at the transcriptional level and exhibits distinct expression reprogramming under PEG-simulated dehydration and salt stress, implying their potential involvement in barley abiotic stress responses.

## Introduction

*H. vulgare* (*Hordeum vulgare* L.) ranks as the world’s fourth most important cereal crop, serving as a vital source for food, animal feed, and as a core raw material for the brewing industry (Jiang et al., 2025). However, its global cultivation is severely constrained by abiotic stresses, particularly drought and soil salinity, which threaten both yield stability and grain quality (Xiong et al., 2025). Consequently, elucidating the molecular mechanisms underlying *H. vulgare* response to abiotic stresses and identifying key stress-tolerance genes are of significant theoretical and practical importance for developing new *H. vulgare* cultivars with high yield, stability, and enhanced stress resilience.

The root system is not only the primary organ for water and nutrient uptake and anchorage but also the first line of defense in sensing and responding to soil environmental stresses (Broadley et al., 2003; Geldner, 2013; Lynch, 2013). Central to this defensive role is the endodermis, where the Casparian strip-a hydrophobic band composed of lignin and suberin-forms a precise apoplastic barrier that selectively regulates the flow of water and solutes (Naseer et al., 2012; Geldner, 2013; Barberon et al., 2016; Wang et al., 2019). Recent studies have identified Casparian strip membrane domain proteins (CASPs) and their more extensive homologs in monocots, known as CASP-like proteins (CASPLs), as the core components constituting and maintaining this functional membrane domain (Roppolo et al., 2011). In *A. thaliana* (*Arabidopsis thaliana*), AtCASP1-5 assemble into a stable protein complex at the plasma membrane, providing a spatial scaffold for the localized deposition of cell wall materials essential for barrier integrity (Roppolo et al., 2011).

Recent studies have revealed that the *CASPL* gene family is also involved in the responses of plants to various abiotic stresses. For instance, in *O. sativa* (*Oryza sativa*), several *OsCASPL* genes are significantly upregulated under drought and salt treatments (Xue et al., 2024a). In *Z. mays* (*Zea mays*), a genome-wide analysis identified 47 *ZmCASPL* members with expression profiles indicative of broad drought and salt stresses adaptation (Xue et al., 2024b). In *C. lanatus* (*Citrullus lanatus*), the cold-inducible gene *ClCASPL* and its *A. thaliana* ortholog *AtCASPL4C1* have been shown to negatively regulate plant growth and cold tolerance, revealing a novel role of *CASPL* members in cold stress response (Yang et al., 2015). In *Z. mays*, the CASPL-encoding gene *ZmSRL5* modulates cuticular wax morphogenesis and enhances drought tolerance by sustaining normal wax layer structure and function (Pan et al., 2020). In *G. hirsutum* (*Gossypium hirsutum*), *GhCASPL1* facilitates secondary cell wall thickening of *G. hirsutum* fibers by stabilizing plasma membrane-localized cellulose synthase complexes, highlighting its regulatory role in cell wall modification (Zhang et al., 2024). In addition, recent studies have updated the stress-related functions of crop *CASPL* genes: *OsCASP1* negatively modulates salt sensitivity and leaf senescence via regulating suberin deposition in roots (Yang et al., 2026).

We note that despite extensive studies of *CASPL* genes in other plant species, there has been no published systematic genome-wide analysis for barley *HvCASP/CASPL* family members. This study aims to perform a comprehensive genome-wide identification and bioinformatics analysis of the *CASPL* gene family in *H. vulgare* and to investigate its expression regulation under abiotic stress. The specific research objectives were as follows: (1) to systematically identify all *HvCASPL* family members, and comprehensively characterize their gene structures, phylogenetic relationships, and chromosomal distribution patterns; (2) to explore the gene expansion mechanisms and adaptive selection pressures driving the evolution of the *HvCASPL* family; (3) to analyze the composition and potential functions of cis-acting regulatory elements in the promoter regions of *HvCASPL* genes; and (4) to reveal the tissue-specific expression patterns of *HvCASPL* genes and their dynamic responses to ABA, drought and salt stress. The findings of this study will provide the first systematic elucidation of the composition and characteristics of the *H. vulgare CASPL* gene family and offer valuable genetic resources and a theoretical foundation for identifying key candidate genes involved in *H. vulgare* abiotic stress responses.

## Materials and methods

### Genome-wide identification of the *CASPL* gene family in *H. vulgare*

The protein sequence file, GFF and genome file of *H. vulgare* were downloaded from the Phytozome 13 (https://phytozome-next.jgi.doe.gov/) (Goodstein et al., 2012). AtCASPL and OsCASPLs protein sequences were downloaded from the TAIR (https://www.arabidopsis.org/) and Rice Genome Annotation Project (https://rice.uga.edu/) databases, respectively. To ensure comprehensive and accurate identification of the *HvCASPL* family, a dual-strategy approach combining homology searching and domain validation was employed. First, a Hidden Markov Model (HMM) search was colnducted using HMMER (Potter et al., 2018). The conserved CASPL domain profile (Pfam: PF04535) was utilized to scan the *H. vulgare* protein sequences with an E-value cutoff of 1e^-5^. Second, BLASTP searches were performed using the AtCASPL and OsCASPL protein sequences as queries against the *H. vulgare* protein database, retaining hits with an E-value ≤ 1e^-5^. Candidate sequences obtained from both methods were merged. Subsequently, the conserved domains of all candidate proteins were verified using the Pfam (http://pfam.xfam.org/) (Mistry et al., 2021) and NCBI-CDD (https://www.ncbi.nlm.nih.gov/Structure/bwrpsb/bwrpsb.cgi) databases. The Pfam database served as the primary screening criterion: only proteins possessing the complete PF04535 (DUF588, CASPL) domain were retained, whereas sequences harboring truncated or partial PF04535 domains were eliminated. Domain architectures of the candidates were further cross-validated using the NCBI-CDD database. Notably, CASPL proteins belong to the MARVEL-domain superfamily. Within this superfamily, plant members are annotated as PF04535 (DUF588, CASPL) in Pfam, while animal homologs are designated MARVEL (PF01284). Therefore, NCBI-CDD may assign the higher-order “MARVEL superfamily” annotation to plant proteins carrying PF04535. This superfamily level label does not indicate the presence of animal-type MARVEL (PF01284) domains or loss of the PF04535 signature. Finally, 36 non-redundant HvCASP/CASPL members were obtained for subsequent analyses. The nomenclature of barley CASP/CASPL family members was established according to their homology to the functionally characterized canonical *CASP* genes in *A. thaliana* (*AtCASP1-AtCASP5*) (Roppolo et al., 2011) and *O. sativa* (*OsCASP1-OsCASP5*) (Wang et al., 2019). Among the 36 identified barley members, three genes exhibiting the highest sequence similarity to these functionally validated canonical CASP orthologs were designated *HvCASP1-HvCASP3*. The CASPL (CASP-like) family, however, constitutes a large protein family in plants, and except for the canonical CASP members mentioned above, the majority of CASPL homologs have neither experimentally verified functions nor standardized gene names in public databases. Therefore, the remaining 33 divergent barley homologs, which belong to the CASP-like clade, were named *HvCASPL1-HvCASPL33* according to their physical order along *H. vulgare* chromosomes. This nomenclature scheme distinguishes the functionally characterized canonical CASP members from the uncharacterized CASP-like paralogs and avoids potential confusion with previously reported genes. Finally, the basic physicochemical properties of all identified members, including amino acid number, molecular weight (MW), and isoelectric point (pI), were predicted using the Tbtools software (Chen et al., 2023).

### Phylogenetic, gene structure, and conserved motif analyses

Multiple sequence alignment of CASPL proteins from *H. vulgare*, *A. thaliana* and *O. sativa* was performed using ClustalW. A phylogenetic tree was constructed using the Neighbor-Joining (NJ) method in MEGA 7.0 (Kumar et al., 2016) software with the following parameters: 1000 bootstrap replicates.

The exon-intron structure of *HvCASPL* genes was visualized using the GFF3 annotation file and the “Gene Structure View” function in TBtools-II software (Chen et al., 2023).

The conserved motifs in HvCASPL proteins were analyzed using the online MEME suite (http://meme-suite.org/) with parameters set as follows: maximum number of motifs = 10, motif width = 6 to 50 amino acids.

### Duplication events and Ka/Ks analysis

The physical positions of *HvCASPL* genes on chromosomes were determined based on genome annotation information, and a chromosomal distribution map was generated using TBtools (Chen et al., 2023). Synteny analysis within the *H. vulgare* genome was performed using MCScan X software to identify syntenic blocks resulting from tandem duplication (TD) and whole-genome duplication (WGD) or segmental duplication events (Wang et al., 2012).

The TBtools (default parameters) were used to analyze ka, ks and Ka/Ks ratios (Chen et al., 2023).

Collinear relationships between *H. vulgare* and four representative species (*A. thaliana*, *S. bicolor*, *O. sativa* and *Z. mays*) were analyzed using Tbtools-II software (Chen et al., 2023).

### Promoter cis-acting element analysis

A 2000 bp sequence upstream of the start codon (ATG) of *HvCASPL* genes was extracted from the *H. vulgare* genome as the putative promoter region. The PlantCARE online database (http://bioinformatics.psb.ugent.be/webtools/plantcare/html/) was used to predict cis-acting regulatory elements within these promoter sequences.

### Expression pattern analysis

Publicly available RNA-seq expression data (FPKM values) for *HvCASPL* genes in various tissues (e.g., root, stem, leaf, internode, young spike, seed) were downloaded from the BarleyExpDB database (http://barleyexp.com/). The sampled tissues and their corresponding developmental stages were as follows: 4-day embryos dissected from germinating grains, Roots from the seedlings (10 cm shoot stage), Shoots from the seedlings (10 cm shoot stage), Etiolated (10 day old seedling), Developing tillers at six leaf stage, 3rd internode, Root (4 weeks), Epidermis (4 weeks), Developing inflorescences (1-1.5 cm), Young developing inflorescences (5mm), Rachis (5 weeks PA), Lemma (6 weeks PA), Lodicule (6 weeks PA), Palea (6 weeks PA), Senescing leaf (2months), Developing grain, bracts removed (5DPA) and Developing grain, bracts removed (15 DPA). The RNA-seq dataset accession numbers: PRJEB14349. Expression heatmaps were generated using the “Heatmap Illustrator” function in TBtools. FPKM values were normalized by Row Scale.

### Plant materials and PEG and NaCl treatment

*H. vulgare* (cv. Morex) seeds were germinated and hydroponically cultivated in 1/5-strength Hoagland solution for 12 days until seedlings reached the three-leaf stage. Seedlings were grown in a growth chamber under a 12 h light/dark cycle at 25 °C with 70% relative humidity. For salt stress (200 mM NaCl), osmotic/drought stress (20% w/v PEG 8000), and ABA treatment (100 μM ABA) were applied by supplementing the corresponding reagents into fresh 1/5-strength Hoagland nutrient solution (Ren et al., 2025). Seedlings maintained in standard 1/5-strength Hoagland solution served as parallel untreated controls.

These treatment concentrations were selected according to published barley stress studies and our preliminary assays: 200 mM NaCl is widely used to induce sublethal salt-stress responses in barley seedlings; 20% (w/v) PEG 8000 was applied to mimic osmotic-drought stress; and 100 μM ABA is commonly adopted to activate ABA-dependent signaling in cereal seedlings. Under these conditions, seedlings exhibited typical stress-related phenotypes but remained viable throughout sampling, confirming the concentrations were effective and non-lethal. Chemicals including NaCl, PEG 8000 and abscisic acid (ABA) were purchased from (Sigma-Aldrich, USA).

Root tissues were harvested as experimental materials. For NaCl and PEG 8000 treatments, root samples were collected at 24 h and 48 h post-treatment. For ABA treatment, root tissues were sampled at 1 h, 6 h, and 12 h after treatment. Three independent biological replicates were collected for each time-point; harvested roots were immediately frozen in liquid nitrogen and stored at −80 °C prior to total RNA extraction.

### Total RNA extraction and cDNA synthesis

Total RNA was extracted from treated root samples using EasyPure^®^ Universal Plant Total RNA Kit (TransGen Biotech, China), strictly following the manufacturer’s instructions. First-strand cDNA was synthesized from total RNA using the PrimeScript™ RT reagent Kit (Takara, Japan) with gDNA Eraser, strictly following the manufacturer’s instructions.

### Quantitative real-time PCR analysis

Gene-specific primers for the selected *HvCASPL* genes were designed using Primer Premier 5.0 software (**Supplementary Table 1**). RT-qPCR was performed using ChamQ SYBR Color qPCR Master Mix (Vazyme, China) following the manufacturer’s instructions. Briefly, the 10 μL reaction system contained 5 μL 2×SYBR mix, 0.5 μL forward primer (10 μM), 0.5 μL reverse primer (10 μM), 1 μL cDNA template and 3 μL nuclease-free water. The thermal cycling program was as follows: 95 °C for 30 s; followed by 40 cycles of 95 °C for 5 s and 60 °C for 30 s. RT-qPCR reactions were performed on a CFX96 Touch Real-Time PCR Detection System (Bio-Rad, USA). Three technical replicates were included for each reaction. The relative expression levels of *HvCASPL* genes were calculated using the 2^-ΔΔCt^ method, with wheat Actin gene used as the internal reference. The expression stability of *HvActin* across PEG 8000, NaCl and ABA treatments was validated across all time-point samples; no significant shifts in reference-gene Ct values were observed under the applied stress conditions. For all 14 genes subjected to RT-qPCR validation, gene expression at 0 h (untreated control) was uniformly normalized to 1, and expression values at subsequent time-points were calculated relative to the 0-h baseline. This uniform normalization strategy allows direct visualization of fold-change responses upon stress exposure for every tested gene. Raw Ct values of both target genes and the reference gene are provided in **Supplementary Table 3**. Data were statistically analyzed and graphically presented using GraphPad Prism 9.0 software.

## Results

### Genome-wide identification of *CASPL* genes in *H. vulgare*

A total of 36 *CASPL* genes were identified in the *H. vulgare* genome using HMMER and BLASTP. These genes were unevenly distributed across 8 chromosomes, with the highest density observed on chromosome 1 and 3. The naming of *HvCASP1* to *HvCASP3* members was based on their sequence similarity to homologous genes in *O. sativa* and *A. thaliana* (**Figure 1**). This naming strategy not only ensures a systematic comparison with known genes but also provides important reference points for subsequent functional studies. The remaining members were assigned names according to their positions on the *H. vulgare* chromosomes (*HvCASPL1*-*HvCASPL33*).

**Figure 1:**
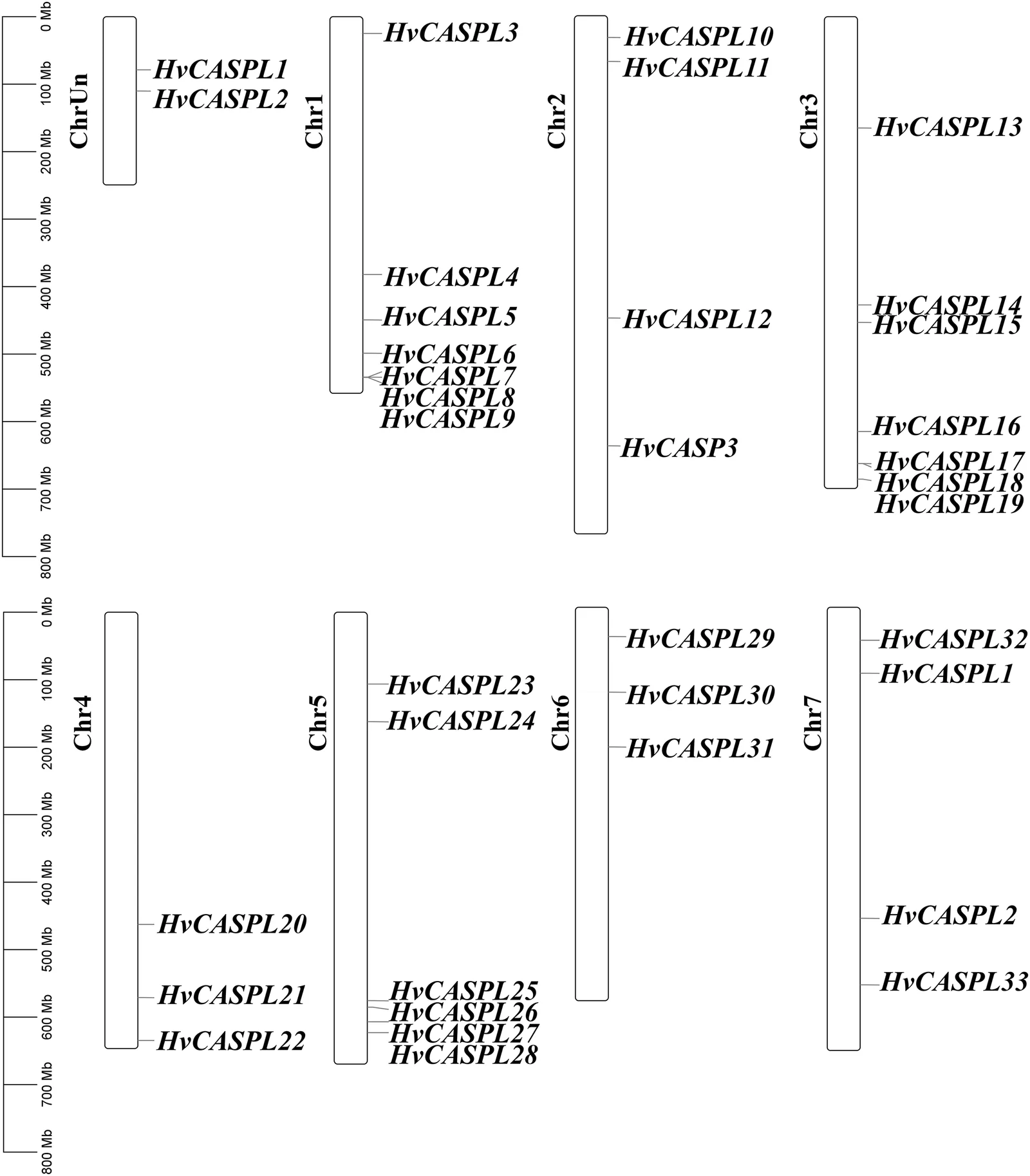
Chromosomal distribution of the *HvCASPL* family genes in *H. vulgare*.

To elucidate the biological characteristics of the *H. vulgare* CASPL family proteins, the physicochemical properties of the 36 identified HvCASPL proteins were systematically analyzed (**Figure 2**; **Supplementary Table 2**). The results revealed considerable variation in protein size, with amino acid lengths spanning from 154 residues in HvCASPL6 to 413 residues in HvCASPL15 (**Figure 2A**). Correspondingly, the predicted molecular weights ranged from 16.00 kDa (HvCASPL13) to 42.89 kDa (HvCASPL14), with a mean value of 23.42 kDa (**Figure 2B**). In terms of isoelectric point distribution, approximately 28% of the family members exhibited an isoelectric point below 7, while 72% had an isoelectric point greater than 7 (**Figure 2C**). Stability assessment via the instability index revealed an even distribution, with 50% of the proteins classified as stable (index < 40) and the remaining 50% as unstable (index > 40) (**Figure 2D**). Additionally, the aliphatic amino acid indices for 83% of the family members ranged between 84.81 and 124.57, reflecting moderate thermostability (**Figure 2E**). Furthermore, the grand average of hydropathicity (GRAVY) indicated that most HvCASPL proteins (30 out of 36 members, 83.33%) were hydrophobic, with a grand average of hydropathicity (GRAVY) value > 0; the remaining six members exhibited GRAVY < 0 and were classified as hydrophilic proteins. (**Figure 2F**). These findings provide valuable insights into the structural and functional properties of the *CASPL* gene family in *H. vulgare*.

**Figure 2:**
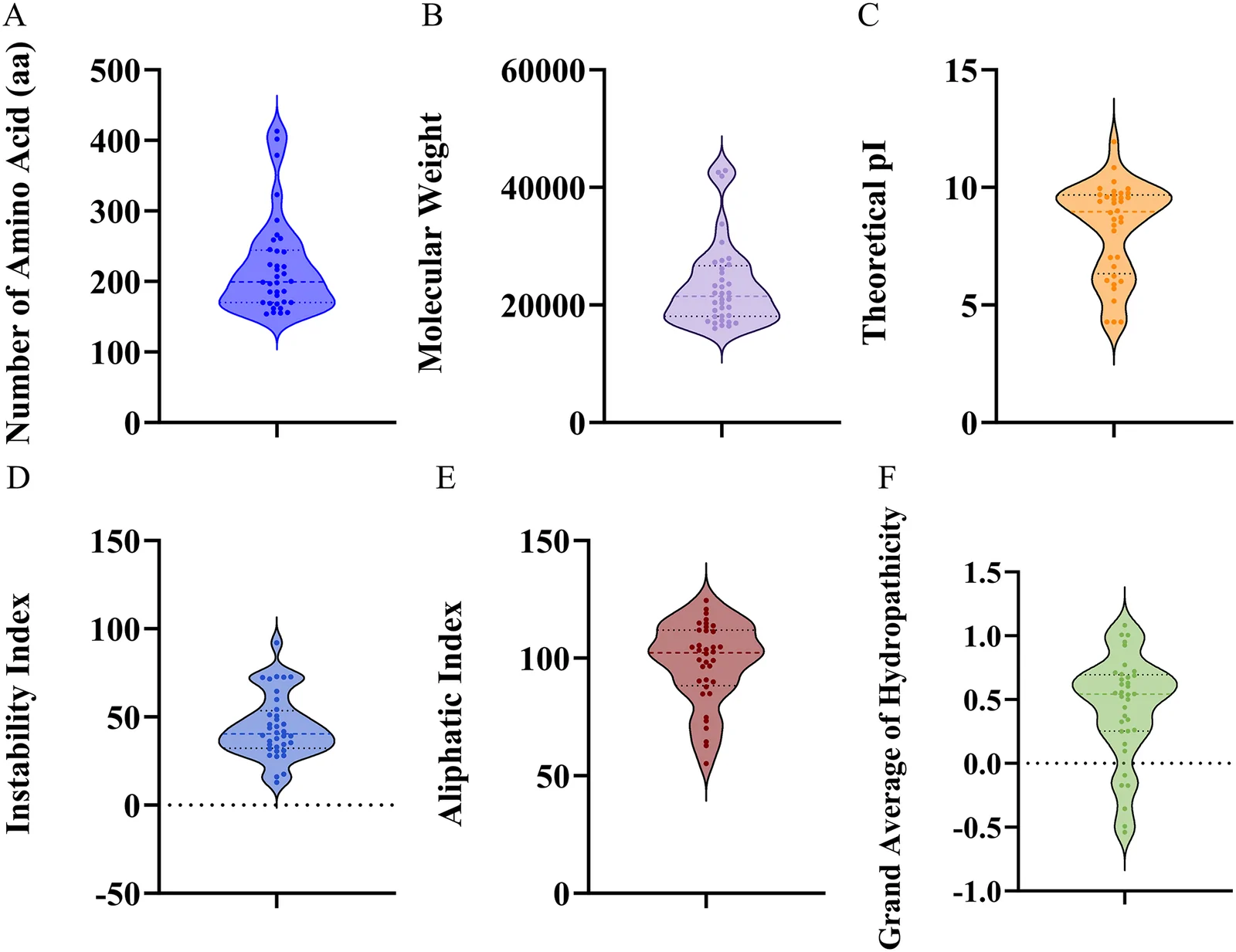
Physicochemical properties of HvCASPL proteins. **(A)** Amino acid sequence length distribution of *H. vulgare* CASPL proteins. **(B)** Molecular weight (MW) distribution. **(C)** Theoretical isoelectric point (pI) values. **(D)** Instability index. **(E)** Aliphatic index. **(F)** Grand average of hydropathicity (GRAVY) values.

### Phylogenetic analysis of CASPL proteins

To elucidate the evolutionary relationships of the CASPL family, a phylogenetic tree was constructed using the full-length amino acid sequences of 36 HvCASPL, 39 AtCASPL and 41 OsCASPL (Xue et al., 2024a) protein sequences. Multiple sequence alignments were performed using ClustalW, and a neighbor-joining (NJ) tree was generated with MEGA 7.0 using 1,000 bootstrap replicates (Kumar et al., 2016). Phylogenetic classification showed that the 116 CASPL proteins were grouped into five Groups I, II, III (subdivided into III-a, III-b, and III-c), IV, and V (**Figure 3**).

**Figure 3:**
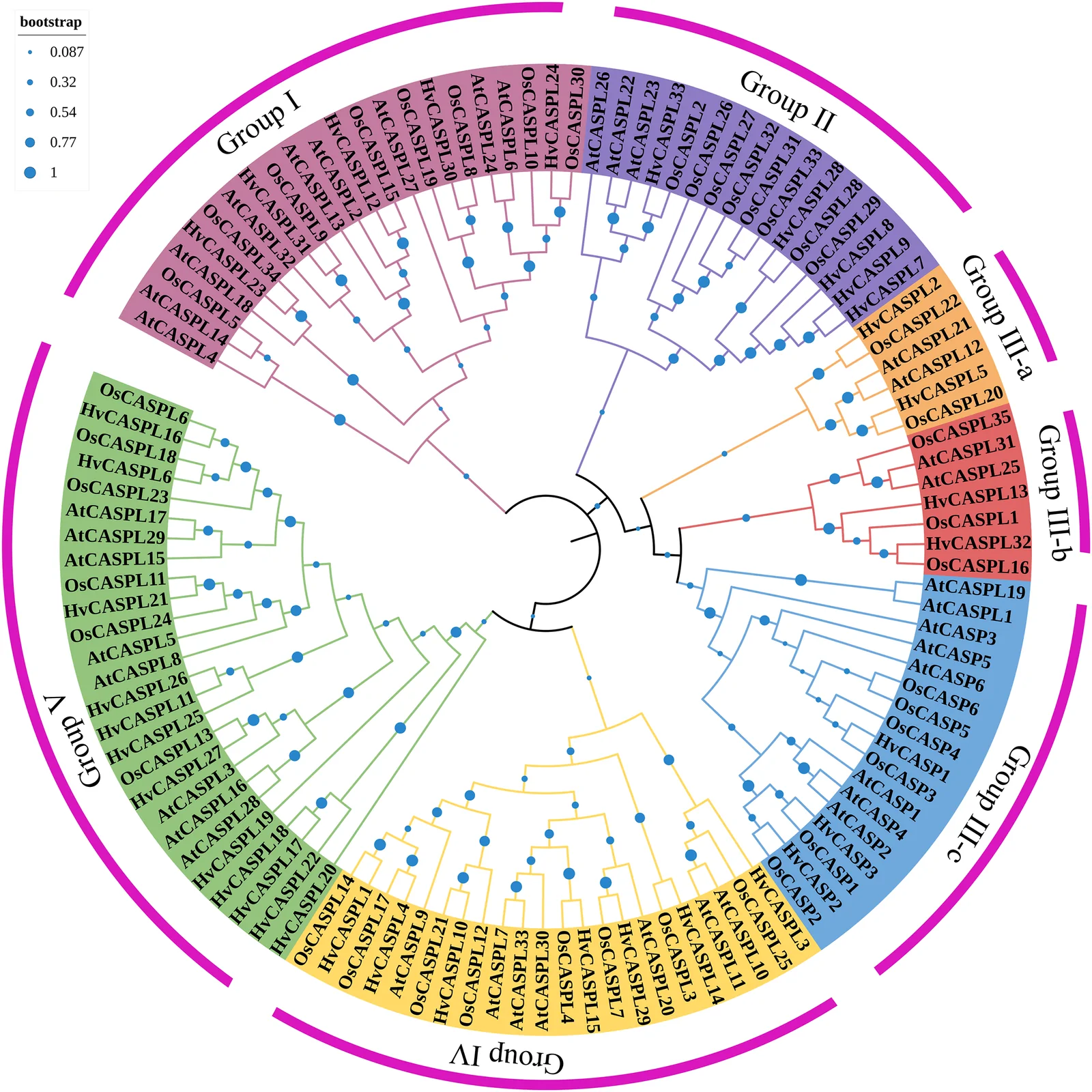
The phylogenetic trees were generated of 36 HvCASPL, 39 AtCASPL and 41 OsCASPL proteins by MEGA 7 (method: Neighbor-Joining; parameter: bootstrap values of 1000 replicates). Different colors represent different Groups (Group I, Group II, Group III, Group IV, and Group V).

Further statistical analysis of the number of CASPL members in each Group showed significant differences in the distribution of members among the five Groups. Among them, Group III contained the largest number of CASPL members, including 12 AtCASPLs, 11 OsCASPLs, and 7 HvCASPLs, accounting for approximately 25.86% of the total 116 CASPL proteins. This result implied that Group III might be the most conserved and functionally important subfamily during the evolution of CASPL proteins, possibly involved in core biological processes shared by monocots and dicots. The second largest subfamily was Group V, which contained 8 AtCASPLs, 6 OsCASPLs, and 12 HvCASPLs, followed by Group I (9 AtCASPLs, 8 OsCASPLs, 5 HvCASPLs) and Group IV (7 AtCASPLs, 8 OsCASPLs, 7 HvCASPLs), Group II (3 AtCASPLs, 8 OsCASPLs, 5 HvCASPLs).

Moreover, the distribution of CASPL members from monocots (*H. vulgare* and *O. sativa*) and dicots (*A. thaliana*) within each subfamily demonstrated a degree of conservation as well as species specificity. For instance, Group II contained fewer AtCASPL members but relatively more HvCASPL and OsCASPL members, suggesting that these subfamilies may have undergone functional expansion in monocot crops, potentially related to their distinct growth characteristics and environmental adaptations. In contrast, Group IV exhibited a more balanced distribution of members among the three species, indicating that these subfamilies might retain conserved functions across different plant lineages. Overall, this phylogenetic analysis provides valuable insights into the evolutionary origins, functional differentiation, and species-specific adaptations of the CASPL protein family.

### Gene structures and conserved motifs

To explore the diversity of the *HvCASPL* gene family during evolutionary processes and clarify the conservation and functional differentiation of its members, the conserved motifs of 36 HvCASPL proteins were analyzed using the MEME online tool (http://meme-suite.org/). A total of 10 distinct conserved motifs were identified and designated as Motif 1 to Motif 10, respectively. Members of Group I–IV were predominantly characterized by the presence of Motifs 4 and 5, whereas Group V members uniformly harbored Motifs 1 and 3 (**Figures 4A, B**). These shared motifs likely represent the core structural signature essential for the fundamental function of CASPL proteins. Detailed characterization further revealed subtle motif variations among individual members. Specifically, Group III proteins contained Motifs 2, 4, 5, and 6; Group II members typically possessed Motifs 4, 5, 7, and 8, with the exception of HvCASPL28 and HvCASPL33, which lacked Motif 8. Most Group I members contained Motif 2, Motif 4 and Motif 5, with the exception of HvCASPL23, while Group V members generally harbored Motifs 1 and 3, except for HvCASPL3, HvCASPL11, and HvCASPL20 (**Figures 4A, B**). The loss of specific conserved motifs in certain paralogs may further facilitate functional divergence during family evolution, allowing different *HvCASPL* members to respond to distinct stress signals and potentially participate in diverse biological processes.

**Figure 4:**
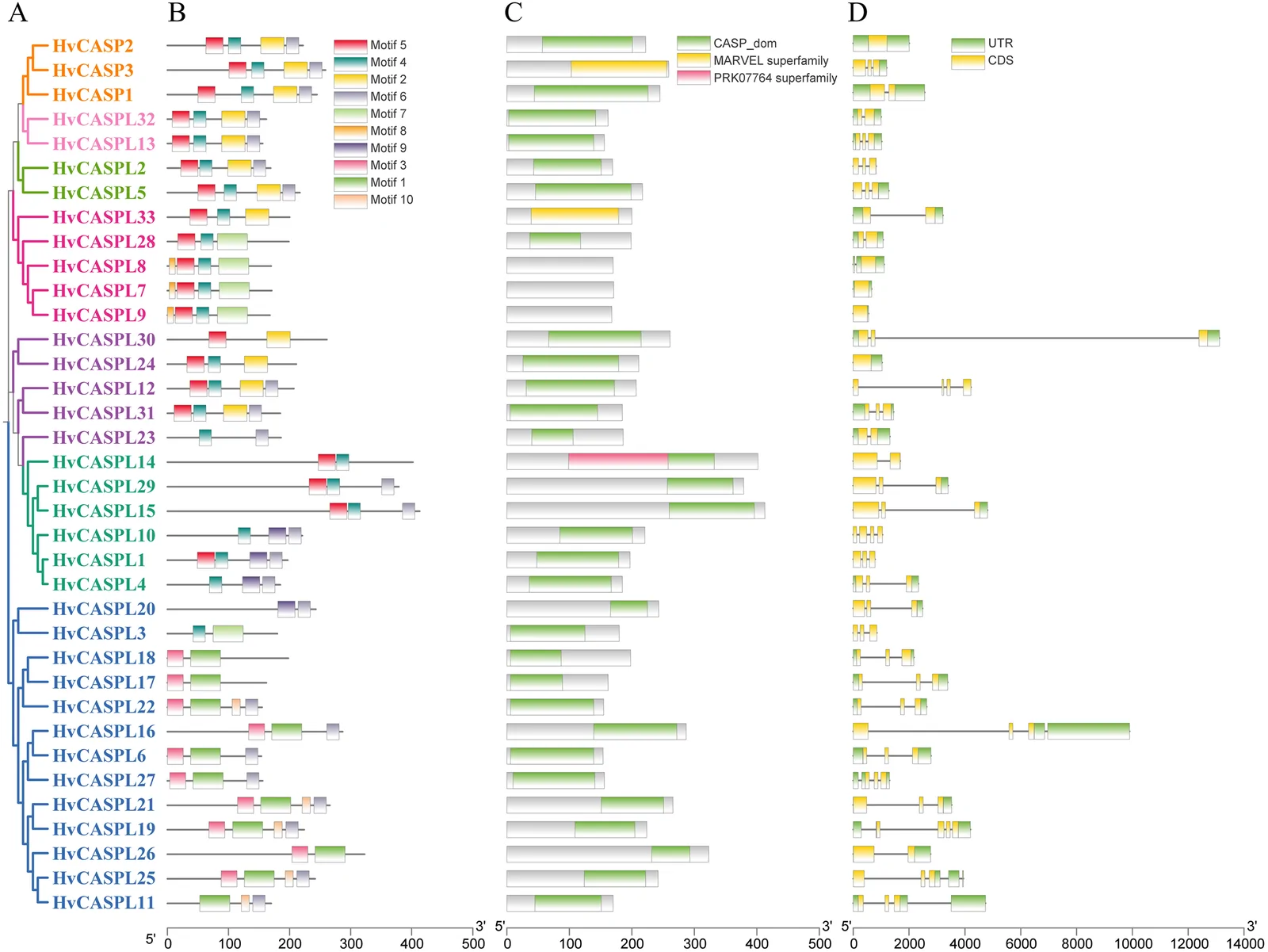
Phylogenetic tree, conserved motif, conserved domain and gene structure were analyzed of HvCASPL. **(A)** Phylogenetic tree of HvCASPL proteins; **(B)** Conserved motifs analysis of HvCASPL proteins. All proteins contain the complete PF04535 (CASPL/DUF588) domain. Two members receive a MARVEL-superfamily annotation in NCBI-CDD due to superfamily-level classification, while their characteristic domain remains CASPL (PF04535). Different colored boxes represent different motifs. **(C)** Schematic diagram of the CASP domain architecture. **(D)** Exon–intron structure of *HvCASPL* genes.

Protein domain analysis based on Pfam and NCBI-CDD showed that all 36 HvCASP/CASPL proteins possessed the canonical complete CASP domain (PF04535, also known as DUF588). According to Pfam annotations, 94% (34/36) of the members carry the direct CASP-domain label. For the remaining two proteins (6%), NCBI-CDD assigned the MARVEL-superfamily label to this identical PF04535 domain. This is purely a database-driven naming difference: these two proteins still contain the typical CASP domain and do not possess animal-type MARVEL domains. To elucidate the gene structural characteristics of *HvCASPL* family members, exon–intron architectures and untranslated region (UTR) distributions were visualized using TBtools software. Comparative analysis revealed substantial structural variation among individual *HvCASPL* genes. Several genes, including *HvCASPL1*, *HvCASPL2*, *HvCASPL3*, *HvCASPL10*, and *HvCASPL14*, lacked both 5’ and 3’ UTRs (**Figure 4D**). In addition, ten genes (*HvCASPL3*, *HvCASPL4*, *HvCASPL5*, *HvCASPL9*, *HvCASPL15*, *HvCASPL16*, *HvCASPL24*, *HvCASPL25*, *HvCASPL26*, and *HvCASPL29*) lacked only the 3’ UTR, while the remaining *HvCASPL* genes contained complete 5’ and 3’ UTR regions. In addition, approximately 17% of *HvCASPL* genes contained one intron, 45% contained two introns, and another 17% contained three introns.

### Gene duplication and ka/ks analysis of *CASPL* genes

The CASPL family is a multigene family, and its expansion and functional differentiation are mainly driven by gene duplication events, which are key evolutionary forces shaping the diversity and complexity of gene families in plants (Xue et al., 2024a, b). To elucidate the evolutionary dynamics of the *HvCASPL* family, intraspecific collinearity analysis was performed using MCScan X. The results revealed that the expansion of the *HvCASPL* family was driven by two primary duplication events: tandem duplication (TD) and whole-genome duplication (WGD). Specifically, we identified two TD gene pairs and two WGD gene pairs in the HvCASP/CASPL family. (**Figure 5A**). Both duplication types contributed equally to the expansion of the *HvCASPL* family, demonstrating that TD and WGD events are the dominant evolutionary forces driving the expansion and functional differentiation of *H. vulgare CASPL* genes.

**Figure 5:**
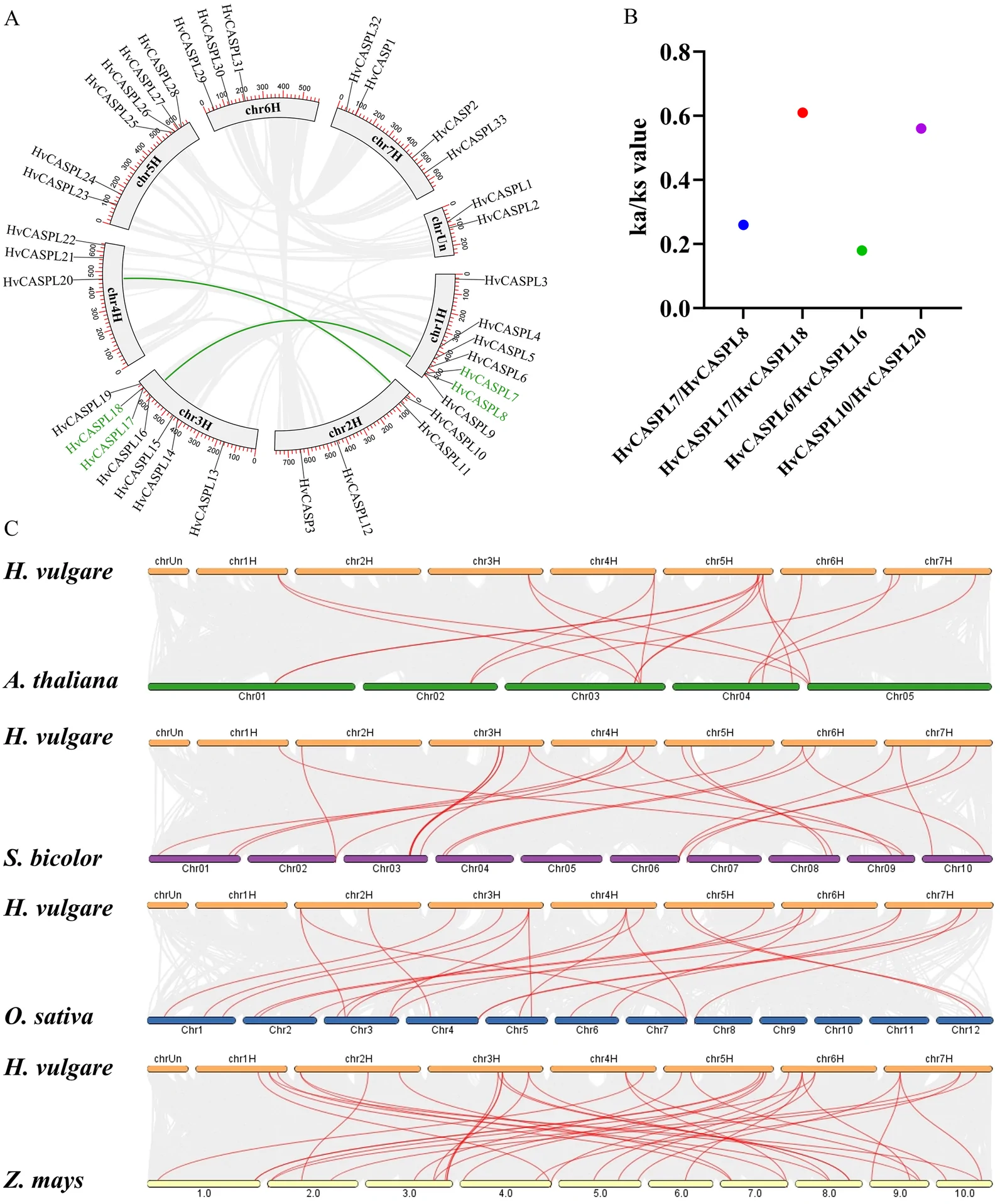
Gene duplication and synteny analysis of the *HvCASPL* gene family. **(A)** Gene duplication events of *HvCASPL* genes. Whole-genome duplication (WGD) events are indicated by green lines, and tandem duplicated genes are labeled with green gene IDs. **(B)** Ka/Ks analysis of *HvCASPL* genes. The ratio of nonsynonymous (Ka) to synonymous (Ks) substitutions was calculated to assess selection pressure. **(C)** Interspecific synteny analysis between *H. vulgare* and *A. thaliana*, *S. bicolor*, *O. sativa* and *Z. mays*.

To further understand the selective constraints acting on duplicated genes, the non-synonymous (Ka) to synonymous (Ks) substitution rate ratios were calculated using KaKs_Calculator. Among these duplicated pairs, two TD and two WGD pairs exhibited Ka/Ks < 1 (**Figure 5B**), indicating that the evolution of the *HvCASPL* family has been governed by strong purifying selection.

Furthermore, interspecific collinearity analysis was conducted to compare the *H. vulgare CASPL* genes with orthologs from *A. thaliana*, *S. bicolor*, *O. sativa* and *Z. mays*. In total, 17, 22, 23, and 36 collinear orthologous gene pairs were detected for *A. thaliana*, *S. bicolor*, *O. sativa* and *Z. mays*, respectively. The results showed extensive collinear relationships between *HvCASPL* and *S. bicolor*, *O. sativa* and *Z. mays CASPL* genes, with numerous orthologous gene pairs identified (**Figure 5C**). In contrast, collinearity between *H. vulgare* and *A. thaliana CASPL* genes was relatively limited. These findings indicate that *H. vulgare* shares a closer evolutionary relationship with monocotyledons *S. bicolor*, *O. sativa* and *Z. mays*, and their *CASPL* homologs may retain conserved biological functions inherited from common ancestral genes. Thus, interspecific collinearity offers a robust framework for annotating *CASPL* gene functions based on the well-characterized orthologs in model plants.

### Analysis of cis-acting regulatory elements in *HvCASPL* gene promoters

To gain insights into the potential regulatory mechanisms, we analyzed the promoter regions (2 kb upstream of the start codon) of *HvCASPL* genes. Cis-element analysis using PlantCARE revealed abundant regulatory elements associated with hormone responses (ABA, auxin, gibberellin, methyl jasmonate), abiotic stress (drought, salt, low temperature), and developmental processes (meristem expression, endosperm development) (**Figure 6**; **Supplementary Table 4**).

**Figure 6:**
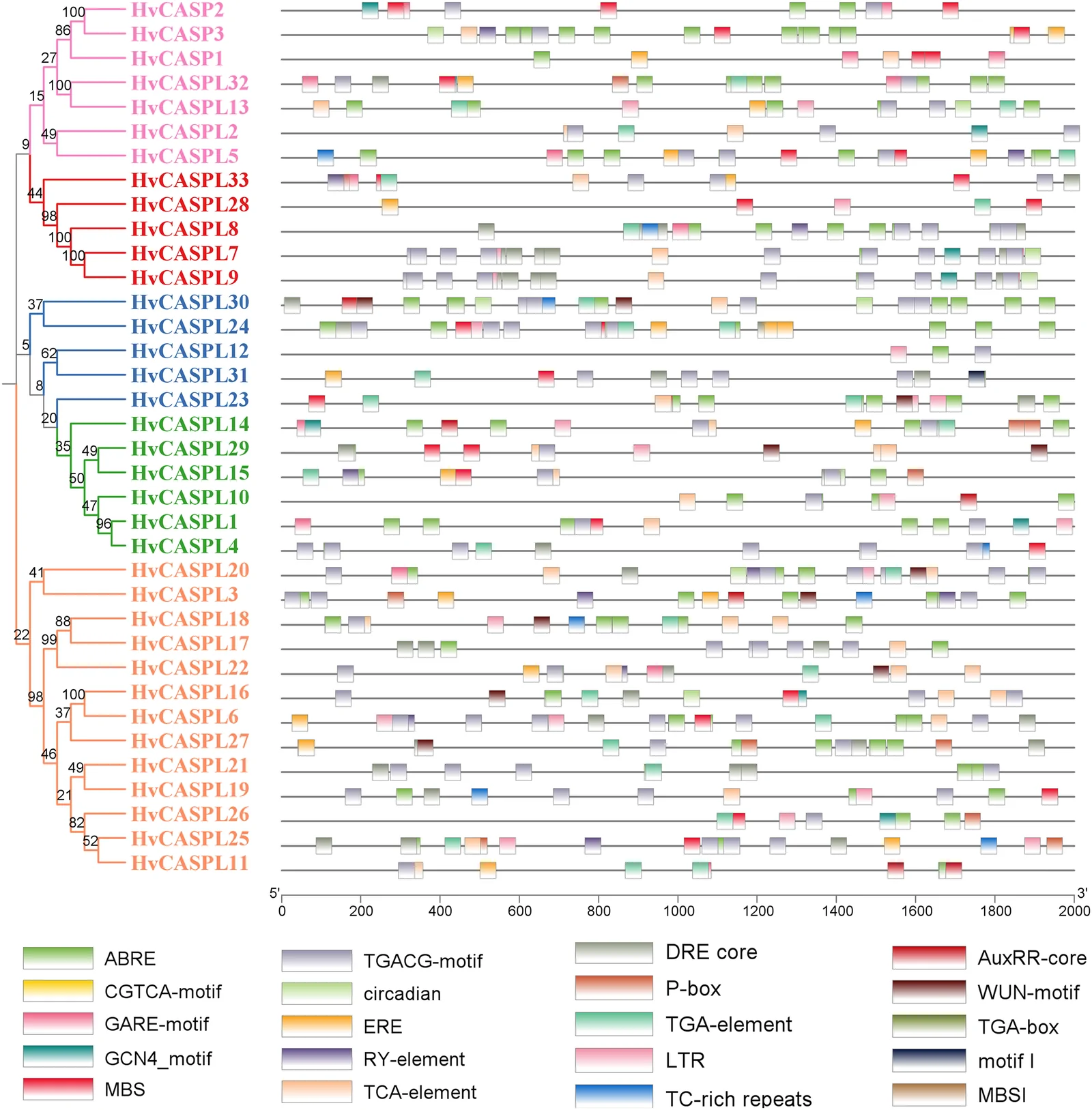
The distribution of major categories of cis-elements across the promoter regions of *HvCASPL* members.

Hormonal regulation appeared to be a dominant feature. Abscisic acid (ABA)-responsive elements (ABREs) were the most prevalent, with particularly high abundance observed in the promoters of *HvCASP3*, *HvCASPL17*, *HvCASPL6*, and *HvCASPL5*. The co-occurrence of ABREs with MYB binding sites and drought-responsive elements (MBS) in these genes suggests their strong involvement in ABA-mediated stress signaling (**Figure 6**). Furthermore, elements responsive to methyl jasmonate (MeJA), gibberellin (GA), auxin, and salicylic acid (SA) were widely distributed. The frequent detection of CGTCA/TGACG motifs (MeJA-responsive) across the majority of promoters implies that the *HvCASPL* family genes (*HvCASPL4*, *HvCASPL7*, *HvCASPL9*, *HvCASPL17* and *HvCASPL20*) is broadly integrated into jasmonate-mediated defense pathways. The co-existence of multiple hormone-responsive elements indicates that the expression of HvCASPL genes is likely governed by a complex network of interacting hormonal signaling pathways. Moreover, the low-temperature responsive element (LTR) was specifically detected in genes including *HvCASPL6*, *HvCASPL13*, *HvCASPL23* and *HvCASPL25*, indicating their potential involvement in cold stress responses. Interestingly, a root-specific regulatory element was uniquely identified in the promoter of *HvCASPL31*, suggesting that this particular gene may play a specialized role in root development or function. In summary, the promoters of the *HvCASPL* gene family are enriched with a diverse array of cis-acting elements associated with hormone signaling, stress responses, and growth/development. The combinatorial differences in regulatory elements among members further suggest the existence of functional specialization and specificity in expression regulation within the family.

### Expression profiling of *HvCASPL* genes in different tissues

To systematically investigate the potential biological functions of the *HvCASPL* family, the expression profiles of *HvCASPL* genes across 16 distinct tissues and developmental stages were analyzed using publicly available transcriptome data. Hierarchical clustering revealed distinct expression patterns, indicating functional divergence within the family (**Figure 7**). Overall, some *HvCASPL* genes displayed pronounced tissue-specific expression, suggesting specialized roles in particular developmental processes. Notably, *HvCASPL4*, *HvCASPL24*, *HvCASPL28* and *HvCASPL32*, were specifically and highly expressed in lemma; *HvCASPL10* and *HvCASPL13* were specifically and highly expressed in rachis. This strong spatial expression pattern implies that these genes may play critical roles in the development of floral organs and spikelet morphogenesis in *H. vulgare*.

**Figure 7:**
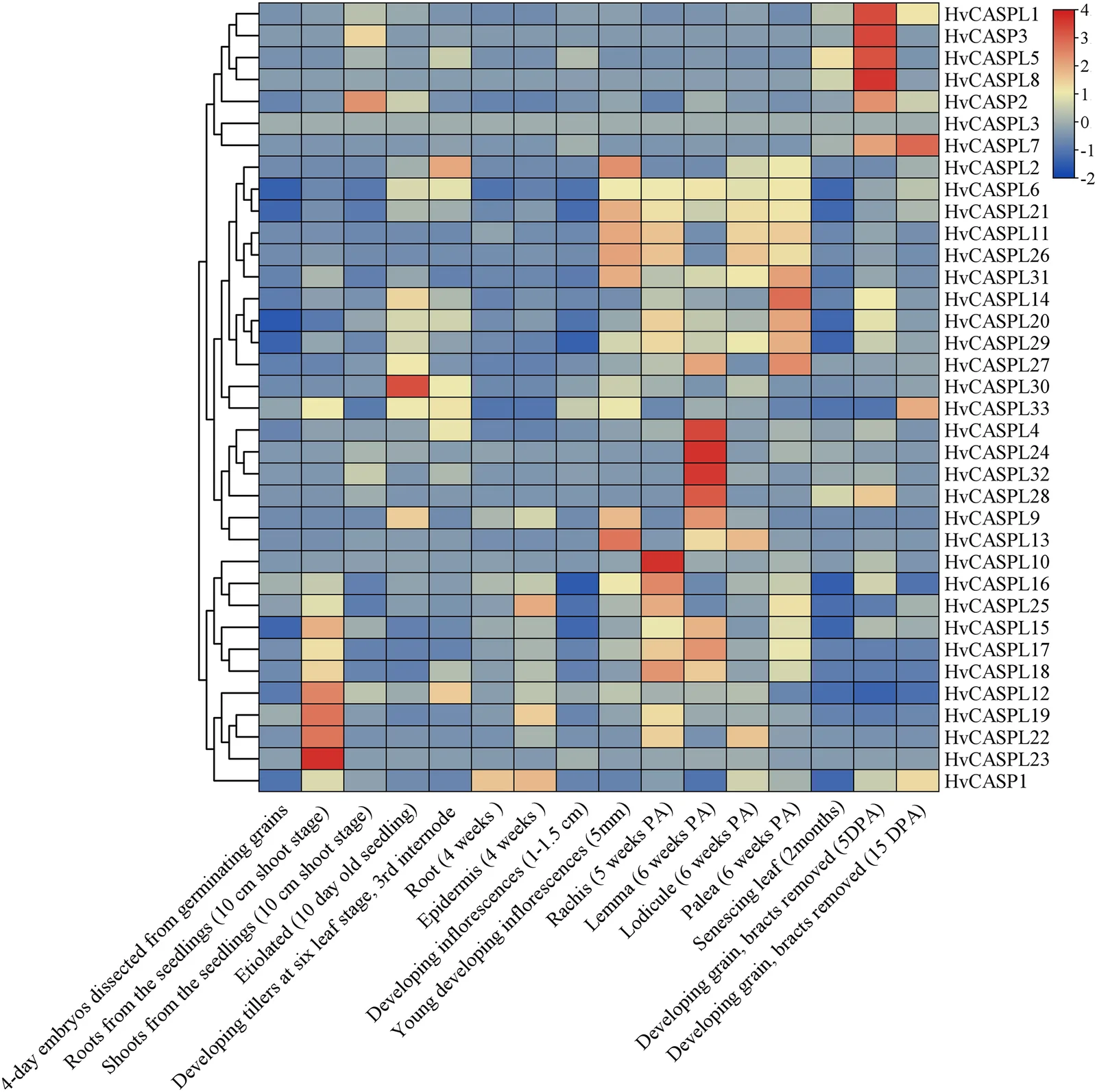
Expression pattern analysis of *HvCASPL* genes in different tissues. The heatmap is constructed by Tbtools software. Red and blue boxes indicate high and low expression levels of *HvCASPL* genes, respectively.

In addition, *HvCASPL1*, *HvCASP3*, *HvCASPL5* and *HvCASPL8* genes exhibited high expression levels in developing grains (bracts removed), indicating that these members may be involved in processes related to grain filling, such as endosperm cell wall modification, membrane microdomain organization, or assimilate transport across the aleurone–endosperm interface. Furthermore, *HvCASPL2* and *HvCASPL7* exhibited high expression levels in developing grains at 15 DPA, suggesting their putative function in grain filling and seed maturation. In contrast, *HvCASPL30* showed preferential expression in etiolated seedlings, implying a possible role in skotomorphogenesis or light signaling regulation. *HvCASPL12*, *HvCASPL19*, *HvCASPL22*, and *HvCASPL23* showed higher expression in roots compared with other tissues. Considering the well-established role of CASPL proteins in assembling the Casparian strip membrane domain in the root endodermis (Wang et al., 2019), it is reasonable to hypothesize that these *HvCASPL* members are involved in the establishment or reinforcement of the endodermal diffusion barrier in *H. vulgare*.

Collectively, these tissue-specific expression profiles provide valuable insights into the functional divergence of *HvCASPL* genes, highlighting their diverse roles in *H. vulgare* growth, development, and stress adaptation.

### Expression pattern analysis of *HvCASPL* genes in ABA

We further quantified the transcript abundance of selected *HvCASPL* family genes in *H. vulgare* seedlings exposed to exogenous ABA treatment at 0 h (untreated control), 1 h, 6 h and 12 h via qRT-PCR (**Figure 8**). Distinct expression patterns were observed among these *HvCASPL* members in response to ABA stimulus. A majority of tested genes exhibited a pronounced upregulation at 1 h or 6 h followed by a gradual decline at 12 h. Specifically, *HvCASPL4*, *HvCASPL5*, *HvCASPL13*, *HvCASPL22* and *HvCASPL25* all reached their maximum relative expression levels at 6 h after ABA application, which were significantly higher than those at the 0 h control and 12 h timepoint. Among them, *HvCASPL16* displayed the strongest ABA-induced induction, with its transcript level peaking at over 12-fold relative to the untreated control. In contrast, several genes including *HvCASPL6*, *HvCASPL7*, *HvCASPL8*, *HvCASPL23*, *HvCASPL24*, *HvCASPL29* and *HvCASPL33* showed down-regulation upon ABA treatment throughout the whole sampling period.

**Figure 8:**
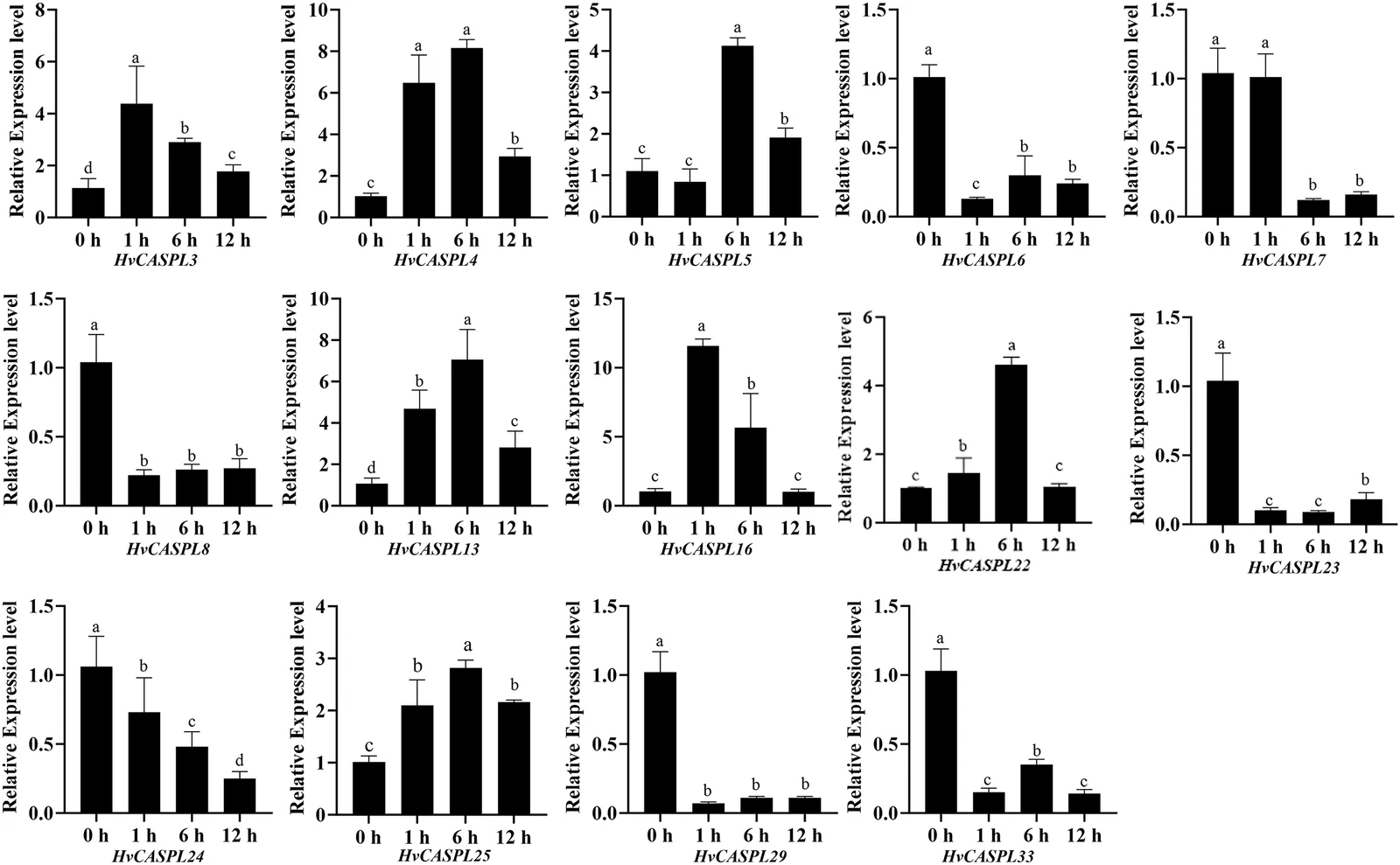
Relative expression profiles of *HvCASPL* genes in *H. vulgare* seedlings under exogenous ABA treatment. Seedling samples were collected at 0 h (untreated control), 1 h, 6 h and 12 h after ABA treatment. The relative expression level at 0 h was set as the reference. Different lowercase letters above bars indicate significant differences between different timepoints (one-way ANOVA, Duncan’s test, p < 0.05). Error bars represent the standard error of three independent biological replicates.

To explore whether the abundance of ABA-responsive cis-elements (ABRE) within promoter regions correlates with transcriptional responses under stress, we compared ABRE counts and RT-qPCR expression profiles for selected *HvCASPL* genes. Genes harboring high numbers of ABRE motifs, such as *HvCASPL3* (7 ABREs), *HvCASPL5* (8 ABREs) and *HvCASPL13* (5 ABREs), exhibited pronounced up-regulation, with expression peaking at 1 h or 6 h after treatment. By contrast, *HvCASPL29* and *HvCASPL33*, which carry only one or zero ABRE motifs, displayed strong transcriptional repression across all time-points (**Figure 9**).

**Figure 9:**
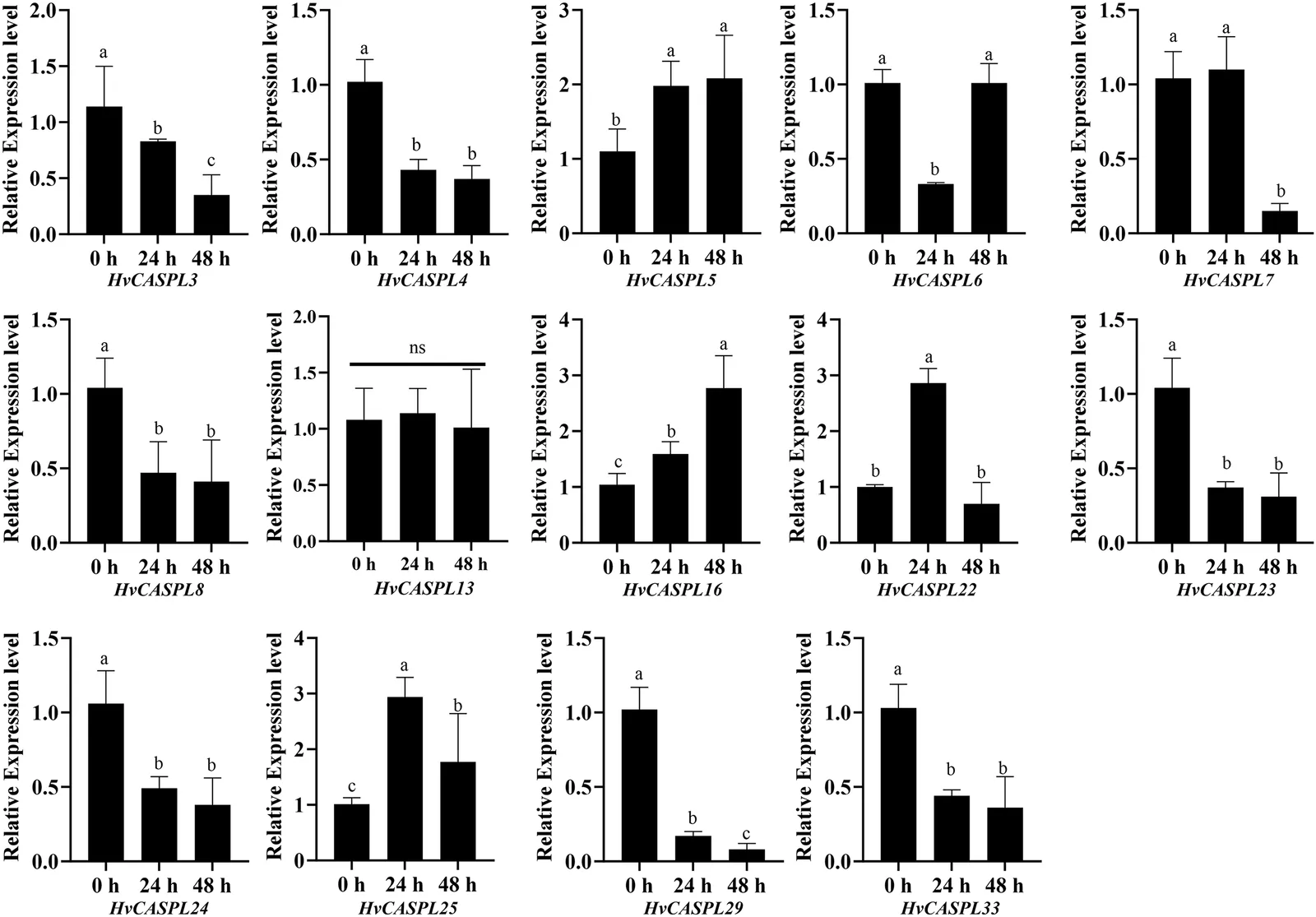
Relative expression patterns of *HvCASPL* genes in *H. vulgare* seedlings under PEG-simulated drought stress. *H. vulgare* seedlings were treated with 20% (w/v) PEG6000 to mimic drought conditions, and samples were collected at 0 h (untreated control), 24 h, and 48 h post-treatment. The relative expression level at 0 h was set as the baseline reference. Different lowercase letters above bars indicate statistically significant differences between time points, and “ns” represents no significant difference (one-way ANOVA followed by Duncan’s test, p < 0.05). Error bars correspond to the standard error of three biological replicates.

Nevertheless, several inconsistencies between ABRE abundance and expression change were also observed. For example, *HvCASPL7* possesses 3 ABRE motifs yet showed obvious suppression, whereas *HvCASPL16*, which contains 2 ABRE copies, was strongly activated. Meanwhile, *HvCASPL8* (7 ABREs) showed rapid transcript decline after 1 h. This observation suggests that ABRE elements may partially contribute to ABA-triggered transcription, but cannot completely account for the observed expression patterns, implying the involvement of other cis-elements or regulatory layers.

### Expression pattern analysis of *HvCASPL* genes in PEG 6000

To explore the transcriptional responses of *HvCASPL* genes to drought stress, we performed qRT-PCR to quantify the relative transcript abundance of 14 *HvCASPL* genes in *H. vulgare* seedlings treated with 20% (w/v) PEG6000. Distinct expression patterns were categorized among the tested gene members (**Figure 9**). A subset of genes exhibited significant induction under PEG-simulated drought stress and reached their peak expression at either 24 h or 48 h. Specifically, *HvCASPL5* maintained consistently elevated transcript levels at 24 h and 48 h. *HvCASPL16* and *HvCASPL25* showed the strongest drought responsiveness: the expression of *HvCASPL16* gradually increased and peaked at 48 h, with its transcript level approximately 4-fold higher than the 0 h control; *HvCASPL25* reached the maximum expression value at 24 h before slightly declining at 48 h, yet remained significantly higher than the untreated group. Additionally, *HvCASPL22* was markedly upregulated at 24 h and decreased to a low level at 48 h. Conversely, most remaining *HvCASPL* genes were continuously suppressed after PEG exposure. For *HvCASPL3*, *HvCASPL4*, *HvCASPL6*, *HvCASPL7*, *HvCASPL8*, *HvCASPL23*, *HvCASPL24*, *HvCASPL29* and *HvCASPL33*, their relative expression levels at both 24 h and 48 h were significantly lower than that of the control. Among these repressed genes, *HvCASPL29* displayed the most dramatic down-regulation, showing extremely low transcript abundance at 48 h.

### Expression pattern analysis of *HvCASPL* genes in NaCl

To characterize the transcriptional responses of HvCASPL family genes to salt stress, qRT-PCR was conducted to detect the relative transcript levels of 14 *HvCASPL* genes in *H. vulgare* seedlings treated with 200 mmol L^-1^ NaCl. Distinct transcriptional patterns were observed across all tested *HvCASPL* members under NaCl stress (**Figure 10**). The vast majority of *HvCASPL* genes were significantly induced by NaCl treatment and exhibited elevated expression at 24 h or 48 h relative to the 0 h control group. Among them, *HvCASPL3*, *HvCASPL4*, *HvCASPL5*, *HvCASPL8*, *HvCASPL13*, *HvCASPL16*, *HvCASPL22*, *HvCASPL23* and *HvCASPL25* displayed a consistent up-regulated trend throughout the treatment period: their transcript abundance gradually increased from 0 h and peaked at either 24 h or 48 h. Notably, *HvCASPL16* showed the strongest salt induction, with its expression level at 24 h reaching more than 10-fold of the untreated control. Several genes including *HvCASPL3*, *HvCASPL4*, *HvCASPL5*, *HvCASPL13* and *HvCASPL25* achieved maximum expression at 48 h, whereas *HvCASPL8*, *HvCASPL16*, *HvCASPL22* and *HvCASPL23* peaked at 24 h before slightly declining at 48 h. In addition, *HvCASPL29* maintained significantly higher expression at both 0 h and 48 h compared with the 24 h treatment group. *HvCASPL33* exhibited decreased expression at 24 h but was strongly induced at 48 h, showing a unique fluctuating expression pattern under salt stress. In contrast, two genes were persistently suppressed upon NaCl exposure. The relative expression of *HvCASPL6* and *HvCASPL7* at 24 h and 48 h was significantly lower than that of the 0 h control, indicating that salt stress repressed their transcription.

**Figure 10:**
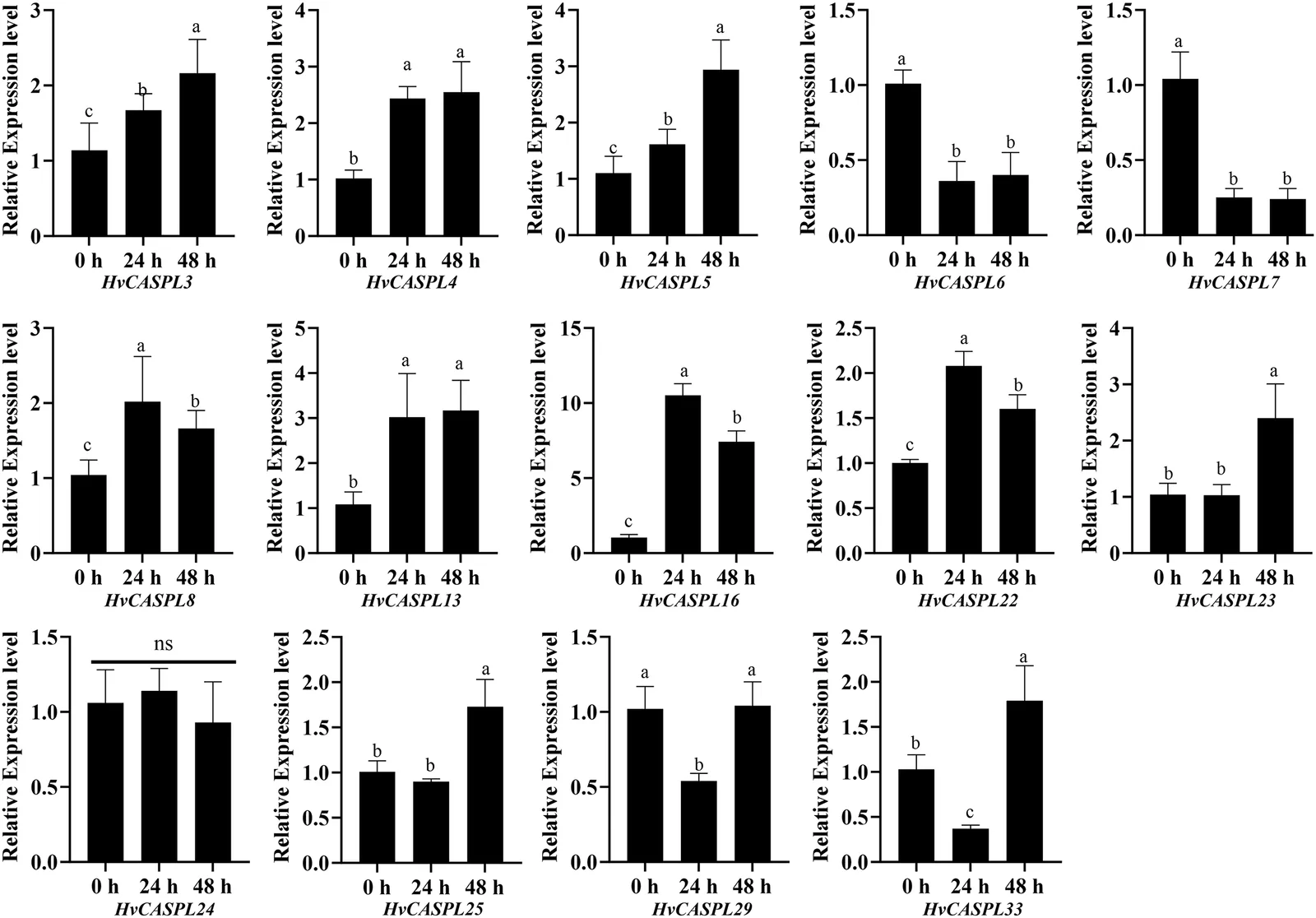
Relative expression profiles of *HvCASPL* genes in *H. vulgare* seedlings under NaCl treatment. *H. vulgare* seedlings were treated with 200 mmol L^-1^ NaCl to simulate salt stress, and seedling samples were collected at 0 h (untreated control), 24 h and 48 h post-treatment. The relative expression value at 0 h was defined as the reference baseline. Different lowercase letters above bars indicate significant differences between sampling time points, and “ns” denotes no significant difference (one-way ANOVA, Duncan’s multiple range test, p < 0.05). Error bars represent the standard error derived from three biological replicates.

Overall, the 14 characterized *HvCASPL* genes exhibited divergent, stimulus-specific transcriptional patterns in response to ABA, NaCl, and PEG treatments. Differential expression profiling revealed that a subset of *HvCASPL* genes were transcriptionally activated under abiotic stress conditions, while others were consistently repressed. These distinct expression signatures reflect transcriptional functional differentiation within the *HvCASPL* family and identify these members as promising candidate genes associated with barley salt and drought stress adaptation. Importantly, all stress-responsive behavior observed here is limited to transcriptional changes, and further genetic evidence is required to confirm their exact biological functions in stress tolerance regulation.

## Discussion

### Genome-wide identification and evolutionary conservation of the *HvCASPL* gene family in *H. vulgare*

As core structural components shaping root endodermal Casparian strips, CASPL proteins construct the apoplastic diffusion barrier and are tightly implicated in plant adaptation to adverse soil environments (Roppolo et al., 2011; Barberon et al., 2016). Comprehensive genome-wide characterizations of the *CASPL* gene family have been documented in the dicot model plant *A. thaliana* and multiple monocot crops, including *O. sativa*, *Z. mays*, *G. hirsutum* and *C. lanatus* (Xue et al., 2024a, b; Yang et al., 2015; Zhang et al., 2024). Nevertheless, systematic profiling of *H. vulgare CASPL* genes and their biological functions remained unreported prior to the present study. Herein, a total of 36 *HvCASPL* genes were identified, which are unevenly distributed across eight *H. vulgare* chromosomes. The gene number of the *H. vulgare CASPL* family is comparable to that of *OsCASPL* (41 members) in *O. sativa* and *AtCASPL* (39 members) in *A. thaliana* (Xue et al., 2024b), indicating that the *CASPL* family has undergone comparable expansion events in both monocot and dicot lineages during long-term plant evolution.

Approximately half of the HvCASPL proteins were predicted to be unstable, which may facilitate dynamic post-translational remodeling of the endodermal barrier under fluctuating abiotic stress conditions. Phylogenetic reconstruction classified all CASPL proteins derived from *H. vulgare*, *O. sativa* and *A. thaliana* into five distinct subfamilies. Among these clades, Group III represents the most evolutionarily conserved subfamily shared by monocots and dicots, implying that genes within this clade retain ancestral core functions indispensable for Casparian strip biogenesis (Naseer et al., 2012). Notably, Group V harbors the largest number of *H. vulgare*-specific paralogs (12 *HvCASPL* genes), indicative of lineage-specific gene expansion that may endow *H. vulgare* with unique adaptive traits to survive drought and salt stresses.

Conserved motif and domain analyses further corroborated the phylogenetic grouping of *HvCASPL* genes. Motifs 4 and 5 constitute the core conserved signatures for Groups I–IV, whereas Motifs 1 and 3 are universally present across Group V members. Partial loss of specific motifs in some HvCASPL represents a common evolutionary strategy to generate subfunctionalized gene copies, a phenomenon also observed in the *O. sativa* and *Z. mays CASPL* families (Xue et al., 2024b). Distinct exon–intron architectures and variable UTR truncations among *HvCASPL* genes further reflect extensive structural remodeling following gene duplication, which frequently reshapes transcriptional responsiveness to environmental stimuli (Pan et al., 2020).

Gene duplication acts as the predominant evolutionary force driving the expansion and functional diversification of multigene families in plants (Magadum et al., 2013). Intraspecific collinearity analysis revealed that both TD and WGD contributed equivalently to the expansion of the *H. vulgare HvCASPL* family. Calculation of Ka/Ks ratios demonstrated that the evolution of the *HvCASPL* family has been constrained by strong purifying selection to maintain conserved protein functions. Consistent selective constraints have been observed in duplicated *CASPL* paralogs from *O. sativa* and *Z. mays* (Xue et al., 2024a, b). Interspecies synteny analysis uncovered extensive collinear relationships between *H. vulgare CASPL* genes and their orthologs from monocots (*S. bicolor*, *O. sativa* and *Z. mays*), whereas collinear orthologous pairs between *H. vulgare* and *A. thaliana* were scarce. This pattern aligns with the deep evolutionary divergence separating monocot and dicot lineages (Lozano et al., 2021; Yang et al., 2023). The conserved syntenic blocks across cereal crops enable translational functional inference: well-characterized stress-related *OsCASPL* and *ZmCASPL* orthologs can serve as reliable references to predict the abiotic stress roles of their corresponding *HvCASPL* counterparts.

### Cis-regulatory landscape and tissue-specific expression profiles uncover multifaceted developmental and stress-related roles of *HvCASPL* genes

Prediction of cis-acting regulatory elements within the 2-kb promoter regions of *HvCASPL* genes via PlantCARE identified abundant motifs associated with phytohormone signaling, abiotic stress responses and developmental processes. ABA-responsive ABRE motifs constituted the most prevalent cis-regulatory feature, consistent with previous findings in *O. sativa* showing that *OsCASPL* promoters are enriched with ABRE motifs and exhibit ABA-inducible transcription (Xue et al., 2024a). The co-occurrence of ABRE and drought-inducible MBS MYB-binding sites in the promoters of *HvCASPL3*, *HvCASPL5*, *HvCASPL6* and *HvCASPL17* suggests that these genes function as central integrators of ABA-dependent drought response cascades. Furthermore, widespread cis-elements responsive to methyl jasmonate (MeJA), gibberellin (GA), auxin and salicylic acid (SA) were detected in *HvCASPL* promoters, indicating that the *HvCASPL* family is integrated into an intricate network of cross-talking phytohormone signaling pathways. A comparable multilayered hormonal regulatory mode has been reported for cotton *GhCASPL* genes that mediate secondary cell wall biosynthesis (Zhang et al., 2024). A unique root-specific cis-element was exclusively detected in the promoter of *HvCASPL31*, providing a molecular basis for its potential specialized function in root development and Casparian strip assembly, consistent with the canonical root-localized functions of CASPL proteins (Geldner, 2013).

Transcriptome-based tissue expression profiling revealed striking spatial expression divergence among *HvCASPL* paralogs, indicative of extensive subfunctionalization governing diverse biological processes throughout *H. vulgare* development. A subset of genes including *HvCASPL12*, *HvCASPL19*, *HvCASPL22* and *HvCASPL23* displayed preferential expression in root tissues, consistent with the well-established function of CASPL proteins in constructing the root endodermal apoplastic barrier (Naseer et al., 2012). Root-enriched *HvCASPL* paralogs are hypothesized to modulate suberin and lignin deposition under salt and drought stress, analogous to *O. sativa OsCASP1*, which negatively modulates salt sensitivity by remodeling root suberin layers (Yang et al., 2026). Several paralogs exhibited exclusive high expression in reproductive organs: *HvCASPL4*, *HvCASPL24*, *HvCASPL28* and *HvCASPL32* accumulated predominantly in lemmas, while *HvCASPL10* and *HvCASPL13* were specifically abundant in rachises. These tissue-restricted expression patterns point to previously uncharacterized roles of CASPL proteins in floral organ differentiation and spikelet morphogenesis, which have rarely been documented in other plant species. Multiple *HvCASPL* genes exhibited robust expression in developing grains, implying their involvement in grain filling, aleurone cell wall modification and photoassimilate transport across the aleurone–endosperm interface. A parallel function in reproductive cell wall modification has been described for cotton *GhCASPL1*, which facilitates secondary cell wall thickening during fiber development (Zhang et al., 2024). Collectively, the divergent tissue-specific expression signatures demonstrate that *H. vulgare HvCASPL* genes have undergone profound subfunctionalization post-duplication, expanding their biological repertoire beyond root Casparian strip formation to orchestrate multiple vegetative and reproductive developmental programs.

### Distinct transcriptional reprogramming of *HvCASPL* genes under ABA, salt and drought stresses reveals functional divergence in abiotic stress adaptation

Exogenous ABA, NaCl and PEG6000 treatments triggered highly divergent transcriptional responses among the 14 selected *HvCASPL* genes, experimentally validating our cis-element prediction that this gene family is tightly regulated by hormonal and abiotic stress signals. ABA acts as the master phytohormone coordinating plant defense against drought and salinity, which activates thousands of stress-mitigating genes via the binding of ABA-responsive transcription factors to ABRE motifs (Tang et al., 2025; Zhang et al., 2025). Upon exogenous ABA application, seven genes (*HvCASPL3*, *HvCASPL4*, *HvCASPL5*, *HvCASPL13*, *HvCASPL16*, *HvCASPL22* and *HvCASPL25*) were significantly upregulated and peaked at 6 h post-treatment, whereas another seven paralogs were persistently suppressed across all sampled time points (1 h, 6 h and 12 h). Among the ABA-inducible genes, *HvCASPL16* exhibited extreme transcriptional induction (>12-fold relative to the untreated control), marking it as a core ABA-responsive *HvCASPL* paralog in *H. vulgare*. This bimodal ABA response pattern mirrors observations in *O. sativa*, where distinct subsets of *OsCASPL* genes are either activated or repressed by exogenous ABA (Xue et al., 2024a), implying antagonistic regulatory roles for different CASPL subclades within ABA signaling cascades.

PEG-simulated dehydration and NaCl triggered salt stress elicited largely overlapping induction profiles for most *HvCASPL* genes. The same set of paralogs induced by ABA were also strongly up-regulated under both drought and salt treatments, among which *HvCASPL16* emerged as the most stress-responsive member across all three experimental conditions (4-fold induction under PEG treatment and >10-fold induction under NaCl exposure). Notably, four members, *HvCASPL5*, *HvCASPL16*, *HvCASPL22* and *HvCASPL25*, were strongly co-up-regulated by ABA, salt and osmotic stress, implying that this group of genes acts as convergent downstream components shared among ABA dependent salt and drought response pathways. To explore the potential regulatory basis underlying their multi-stress responsiveness, we examined cis-regulatory elements within their promoter regions. Promoter analysis revealed that the promoters of *HvCASPL5*, *HvCASPL16*, *HvCASPL22* and *HvCASPL25* harbor multiple ABF-binding ABRE elements (**Figure 6**). The co-occurrence of these stress associated cis elements offers a plausible mechanistic explanation for their simultaneous activation by ABA, salt and drought signals, suggesting that these four genes may be transcriptionally modulated by both ABA dependent and ABA independent stress cascades. Under high salinity conditions, salt inducible *HvCASPL* genes may accelerate suberin deposition in the root endodermis and restrict influx of toxic Na^+^ ions, a function that has been experimentally characterized for rice *OsCASP1* (Yang et al., 2026). Under PEG-mediated dehydration stress, up-regulation of these genes may reinforce Casparian strip integrity to minimize transpirational water loss (Pan et al., 2020). A small subset of paralogs exhibited stress-specific transcriptional behavior: *HvCASPL13* was strongly activated by NaCl but displayed no significant expression fluctuation under PEG-simulated drought, while *HvCASPL24* remained transcriptionally stable under drought, was suppressed by ABA, and showed no response to salt stress. These distinct expression signatures reflect specialized paralog functions tailored to discrete environmental stress stimuli.

Notably, *HvCASPL6* and *HvCASPL7* were consistently down regulated under ABA, PEG induced osmotic and NaCl salt treatments, pointing to their potential roles as negative modulators of abiotic stress tolerance. A comparable repressive function in stress responses has been reported for watermelon *ClCASPL* and its *Arabidopsis* ortholog *AtCASPL4C1*; overexpression of these genes confers reduced cold-stress resistance (Yang et al., 2015). Drawing on this functional precedent, we hypothesize that stress-triggered transcriptional suppression of *HvCASPL6* and *HvCASPL7* may relieve their inhibitory influence over ABA-dependent defense cascades and thereby permit full activation of downstream stress tolerance pathways. This regulatory arrangement could help plants avoid hyper-activation of stress signaling under adverse conditions, which would otherwise incur growth penalties. Nevertheless, this functional model remains inferred solely from gene expression patterns. Further genetic and biochemical assays, such as overexpression and knockout analyses, will be required to experimentally validate the proposed negative regulatory roles of *HvCASPL6* and *HvCASPL7*. Several paralogs exhibited unique fluctuating expression trends under salt stress: *HvCASPL29* was suppressed at 24 h post-NaCl treatment but recovered to high expression levels at 48 h, whereas *HvCASPL33* exhibited reduced expression at 24 h followed by sharp upregulation at 48 h. Such dynamic expression shifts imply delayed transcriptional feedback regulation during prolonged stress exposure. Collectively, these stimulus-specific expression profiles highlight clear functional divergence within the *HvCASPL* family: co-induced paralogs represent high-priority candidate genes for genetic engineering of salt- and drought-tolerant *H. vulgare* varieties, while repressed paralogs may function as negative regulators that can be targeted via gene silencing to enhance crop stress resilience.

It should be noted that high-concentration PEG 8000 generates strong osmotic pressure and may cause non-specific osmotic shock besides mimicking drought stress. Although parallel untreated controls and time-series sampling help differentiate stress-specific transcriptional responses from general osmotic injury, potential contributions of non-specific osmotic effects to gene expression changes cannot be fully excluded. These considerations should be kept in mind when interpreting our RT-qPCR expression data.

## Conclusions and future perspectives

This study provides the first comprehensive genome-wide characterization of the *CASPL* gene family in *H. vulgare*, integrating evolutionary analysis, bioinformatic regulatory prediction and experimental qRT-PCR expression validation to dissect their developmental and stress-related functions. We demonstrate that tandem and whole-genome duplication coupled with purifying selection drove the expansion and functional conservation of the *HvCASPL* family, while subfamily-specific structural motifs and cis-regulatory landscapes enabled widespread subfunctionalization across vegetative/reproductive development and abiotic stress signaling. Stress-induced paralogs such as *HvCASPL16*, which exhibits robust upregulation under ABA, salt and drought treatments, represent high-priority candidate genes for subsequent functional validation. Nevertheless, the present study also has several limitations. First, RT-qPCR analyses were restricted to seedling root samples, and potential expression patterns in other tissues and organs remain to be explored. Second, our analyses are based on transcriptional data, and transcript variations may not reflect protein abundance and physiological consequences. In addition, we have not yet conducted transgenic functional experiments, including gene knockout or over-expression, to verify the biological roles of *HvCASP/CASPL* genes. Future work involving transgenic materials and multi-tissue sampling will help to further unravel the biological functions of *CASPL* gene family in barley.

Future research directions emerging from this work include: (1) subcellular localization assays to verify plasma membrane targeting of stress-responsive HvCASPL proteins; (2) transgenic overexpression and gene knockout of key candidates (e.g., *HvCASPL16*) in *H. vulgare* or *A. thaliana* to genetically confirm their roles in salt and drought tolerance; (3) measurement of root suberin and lignin contents in transgenic lines to uncover the cell wall-modifying mechanisms mediated by stress-induced HvCASPL proteins; (4) yeast two-hybrid screening to identify interacting partners of HvCASPL proteins involved in ABA signal transduction and Casparian strip assembly. The dataset generated in this research lays a foundational theoretical framework and provides valuable genetic resources for molecular breeding of stress-tolerant *H. vulgare* varieties.

## Data Availability

The original contributions presented in the study are publicly available. This data can be found at the European Nucleotide Archive using accession number PRJEB14349 and use hyperlink https://www.ebi.ac.uk/ena/browser/view/PRJEB14349.
